# Confocal Comparison of Corneal Reinnervation after Small Incision Lenticule Extraction (SMILE) and Femtosecond Laser In Situ Keratomileusis (FS-LASIK)

**DOI:** 10.1371/journal.pone.0081435

**Published:** 2013-12-09

**Authors:** Meiyan Li, Lingling Niu, Bing Qin, Zimei Zhou, Katherine Ni, Qihua Le, Jun Xiang, Anji Wei, Weiping Ma, Xingtao Zhou

**Affiliations:** 1 Key Lab of Myopia, Ministry of Health, Department of Ophthalmology, EYE & ENT Hospital of Fudan University, Shanghai, China; 2 Department of Ophthalmology, Bronx Lebanon Hospital Center, Albert Einstein College of Medicine, Bronx, New York, United States of America; 3 Duke University, Durham, North Carolina, United States of America; 4 Department of Biostatistics, School of Public Health, Fudan University, Shanghai, China; UC Berkeley, United States of America

## Abstract

**Purpose:**

To evaluate corneal reinnervation, and the corresponding corneal sensitivity and keratocyte density after small incision lenticule extraction (SMILE) and femtosecond laser in situ keratomileusis (FS-LASIK).

**Methods:**

In this prospective, non-randomized observational study, 18 patients (32 eyes) received SMILE surgery, and 22 patients (42 eyes) received FS-LASIK surgery to correct myopia. The corneal subbasal nerve density and microscopic morphological changes in corneal architecture were evaluated by confocal microscopy prior to surgery and at 1 week, 1 month, 3 months, and 6 months after surgery. A correlation analysis was performed between subbasal corneal nerve density and the corresponding keratocyte density and corneal sensitivity.

**Results:**

The decrease in subbasal nerve density was less severe in SMILE-treated eyes than in FS-LASIK-treated eyes at 1 week (*P = *0.0147), 1 month (*P = *0.0243), and 3 months (*P = *0.0498), but no difference was detected at the 6-month visit (*P* = 0.5277). The subbasal nerve density correlated positively with central corneal sensitivity in both groups (r = 0.416, *P*<0.0001, and r = 0.2567, *P* = 0.0038 for SMILE group and FS-LASIK group, respectively). The SMILE-treated eyes have a lower risk of developing peripheral empty space with epithelial cells filling in (*P* = 0.0005).

**Conclusions:**

The decrease in subbasal nerve fiber density was less severe in the SMILE group than the FS-LASIK group in the first 3 months following the surgeries. The subbasal nerve density was correlated with central corneal sensitivity.

## Introduction

The human cornea is densely innervated and richly supplied by bundled nerve fibers. The corneal nerve fibers and related neurotrophins and neuropeptides play a large role in maintaining homeostasis of the ocular surface. Several neuropeptides and corneal nerve growth factors which regulate corneal epithelial proliferation, integrity, and wound healing, such as substance P and calcitonin gene-related peptide, were shown to be suppressed after refractive surgeries, [1] thus contributing to postoperative corneal hypoesthesia [2], [3], dry eye [4], [5], and LASIK-induced neurotrophic epitheliopathy [6].

Small incision lenticule extraction (SMILE) technology is an all-in-one technology for correcting refractive errors that has become available for intrastromal lenticule cutting and subsequent lenticule extraction. SMILE technology has exhibited excellent efficacy, safety, and predictability [7], [8], [9]. In this flapless SMILE procedure, flaps are replaced with caps, significantly minimizing the incision access to the intrastromal lenticule [7], [8], [9] and, theoretically, transecting fewer superficial nerve fibers [10]. Thus, we hypothesized that the reduction in corneal subbasal nerve density in patients who receive the SMILE procedure would be less severe than the patients who receive femtosecond laser in situ keratomileusis (FS-LASIK).

A recent study [11] reported changes in corneal subbasal nerve morphology and corneal sensation before and 6 months after SMILE and femtosecond lenticule extraction (FLEX). In our study, we used in vivo confocal microscopy to investigate the time-dependent changes (preoperative, postoperative 1 week, 1, 3, and 6 months) in corneal subbasal nerve reinnervation after the SMILE and FS-LASIK procedures to gain a full scope of information on the trends of corneal reinnervation. We also investigated the relationships between subbasal nerve fiber density, keratocyte density and corneal sensitivity.

## Materials and Methods

### Ethics Statement

The Ethical Committee of the Fudan University EENT Hospital Review Board approved the study protocol. The study was conducted in accordance with the principles of the Declaration of Helsinki. Written informed consent was obtained from each participant after the nature of the study had been explained to them. Patients chose their preferred surgical methods after the doctor had explained the nature of the two procedures. The surgeon (X.T.Z) made the decision to treat. All 40 patients in the current study were included in a population of 71 patients in another of our studies which focused on dry eye and corneal sensitivity after SMILE and FS-LASIK (unpublished).

### Patients

Eighteen patients (32 eyes) receiving SMILE and 22 patients (42 eyes) receiving FS-LASIK for the correction of myopia were recruited for the study between June 2010 and March 2012 at the Department of Ophthalmology of Fudan University Eye and ENT Hospital (Shanghai, People’s Republic of China). The same surgeon (X.T.Z.) performed the operations on all of the patients who participated in the study.

All of the patients received a complete ophthalmologic examination before surgery to ensure a normal cornea and anterior segment. The inclusion criteria for both procedures were that patients must be 18 years of age or older, must receive a routine ophthalmic examination, and have a stable refractive error with a minimum calculated residual corneal stromal bed thickness of at least 280 µm.

To reduce possible confounding effects, subjects with any of the following conditions were excluded: (1) subjects using a topical ocular medication on the day of their participation in the study, (2) subjects diagnosed with external ocular disease or who received ocular surgery in the past 6 months, (3) subjects with permanent or temporary lacrimal punctum occlusion, or (4) patients diagnosed with a disease that may affect the ocular surface, such as diabetes or a history of herpetic keratitis.

### Surgical Techniques

The Carl Zeiss Meditec AG VisuMax femtosecond laser system with a repetition rate of 500 kHz and a pulse energy of 130 nJ was used to perform surgical refractive corrections for the patients in the SMILE group. The surgical procedure has been described previously by Sekundo *et al*. [8]. The intended thickness of the upper tissue arcade was 100 µm and the intended diameter was 7.5 mm, which was 1 mm larger than the diameter of the refractive lenticule (6.5 mm). The side cuts made for access to the lenticule were set 90° apart at a circumferential length of 4.5 mm. The refractive lenticule of the intrastromal corneal tissue was dissected through the side-cut opening incision and was manually removed using forceps.

In the FS-LASIK group, flaps were created with a 500-kHz VisuMax femtosecond laser. The flaps had diameters of 8.5 mm, with standard 90° hinges and 90° side-cut angles. The lamellar and side cuts were achieved with energies of 185 nJ. The target flap thickness was 90 µm. The hinges were set in a superior orientation with a hinge length of 4.0 mm. Stromal tissue ablation was performed with the Mel-80 (Carl Zeiss Meditec, Oberkochen, Germany) excimer laser with a repetition rate of 250 kHz and a pulse energy of 150 nJ using a tissue-saving function. All of the procedures were performed under topical anesthesia.

The patients wore bandage soft contact lenses (ACUVE OASYS, Inc., FL, USA) until the day following the operation. The postoperative topical medication regimens were identical for each eye and consisted of the administration of an ophthalmic solution of levofloxacin 4 times per day for 7 days, a 0.1% fluorometholone solution tapered from 8 times per day to 1 times per day over the course of 20 days, and a preservative-free tear supplement (carboxymethylcellulose sodium eye drops, Allergan, Inc., Irvine, CA) 4 times per day for 1 month.

### Corneal Sensitivity

Corneal sensitivity was measured with a Cochet-Bonnet esthesiometer (Luneau, Paris, France) prior to surgery and 1 week, 1 month, 3 months, and 6 months postoperatively. This instrument consists of a nylon monofilament that is 60-mm long and has a diameter of 0.12 mm. The instrument was advanced perpendicularly to the central surface of the cornea until contact between the instrument and the cornea was made. If the patient felt the filament, the response was defined as positive. Corneal sensitivity was tested three times with each filament length, and the length of the filament was sequentially reduced from 60 mm in 5-mm steps. The longest filament length at which at least two positive responses were obtained from three attempts was considered the corneal threshold. All of the measurements were performed under slit-lamp examination by the same observer (M.Y.L.).

### Corneal Innervation and Keratocyte Density

In vivo confocal microscopy (IVCM; HRT III; Heidelberg Engineering, Heidelberg, Germany) was used to evaluate corneal changes. A 60× water-immersion objective lens (Olympus Europa GmbH, Hamburg, Germany) was used with a 670-nm diode laser as a light source, allowing a scanning area of 384×384 pixels covering an area of 400 µm×400 µm with a transverse optical resolution of 2 µm and a longitudinal optical resolution of 4 µm. Before examination, a drop of 0.4% oxybuprocaine hydrochloride (Benoxil; Santen Pharmaceutical Co., Ltd, Osaka, Japan) was delivered to the lower conjunctival sac. At each visit, 3 to 5 scans within the central 3 mm area of the cornea were recorded, and one scan at each of the four regions (superior, inferior, lateral, and nasal) of the cap or flap margins was recorded to observe nerve regrowth and corneal architecture modifications. Nerve fiber bundles were defined as bright, well-defined, linear structures. The process for recording nerve fibers has been described previously by Erie *et al*. [12] Three high quality scans in the central corneal region were selected and measured. The total length of all visible subbasal nerve fibers and branches longer than 50 µm was measured in each scan with Image-Pro version 6.0.0.260 (Media Cybernetics, Inc., Bethesda, USA). The subbasal nerve density for each scan was defined as the total length of the nerves divided by the area of the image and was expressed as micrometers per square millimeter (µm/mm^2^).

Two high quality scans in the central corneal region were selected from the layer in the cap or flap 10 µm anterior to the interface (pre-IF), and two were selected from the layer in the stroma 10 µm posterior to the interface (post-IF). Keratocyte nuclei were manually counted. Keratocyte density was expressed as cells/mm^2^. The thicknesses of the stromal cap/flap and the stromal bed, determined from the 1-month scans, were used to delimit the corresponding pre-IF layer and post-IF layers in the preoperative cornea as described by Lee *et al*. [6]. Subbasal nerve fibers measurement and keratocyte counting were done by the same investigator (L.L.N.), who was blinded to the patient and postoperative time.

### Statistical Analysis

Statistical analyses were performed using SAS 9.3 statistical software (SAS Institute Inc., Cary, North Carolina, USA). The continuous variables were expressed as the mean ± standard deviation (SD), whereas the categorical variables were expressed as the frequency and percentage. The Independent Student’s t-test or Mann Whitney U test was used to compare continuous variables, and the Pearson chi-square test was used to compare the categorical variables at the baseline. A mixed model was constructed to analyse the observations on subbasal corneal nerve density and keratocyte density in both the SMILE group and the FS-LASIK group by taking treated SE at baseline as the selected covariate and taking different times of measurements as the repeated factor. The decreases of the response variables after the surgeries were estimated and compared through the mixed model. Spearman’s rank correlation analysis was used to determine the associations between the subbasal corneal nerve density, keratocyte density and corneal sensitivity.

## Results

### Baseline

The baseline demographic data for the study population is summarized in Table 1. The age, gender, corneal thickness, ablation depth, and corrected distance visual acuity (CDVA) of the two groups were comparable. The preoperative spherical equivalent (SE) (*P*<0.001) and treated SE (*P*<0.001) of the SMILE group were significantly lower than the FS-LASIK group.

**Table 1:** Demographic Data and Preoperative Characteristics.

Characteristics	SMILE group (n = 32 eyes)	FS-LASIK group (n = 42 eyes)	*P* value
Age (mean ± SD, y)	27.1±4.0	28.3±5.5	0.2878
Range of age (y)	20–36	22–39	
Male/Female	9/9	6/16	0.140
Preoperative SE (mean ± SD, D)	−6.56±1.28	−8.46±2.15	<0.001
Range of preoperative SE (D)	−8.25–−4.00	−13.00–−3.30	
Treated SE (mean ± SD, D)	−7.11±1.33	−9.3±2.2	<0.001
Range of treated SE (D)	−9.13–−4.40	−14.0–−3.50	
CCT (µm)	547.34±35.09	545.61±25.67	0.8081
Range of CCT	500–614	500–605	
Ablation depth (mean ± SD, µm)	132.00±22.42	141.83±21.28	0.0598
Range of ablation depth (µm)	92–170	72–168	
CDVA (logMAR)	−0.017±0.040	0.001±0.075	0.2166

SE: spherical equivalent.

CCT: central corneal thickness.

CDVA: corrected distance visual acuity.

### Changes in Corneal Subbasal Nerve Densities

Table 2 contains the changes of subbasal nerve densities over time and a summary of the results from the mixed-model test. The reduction in subbasal nerve density was significantly less in the SMILE-treated eyes than in FS-LASIK-treated eyes (*P = *0.0147, *P = *0.0243, and *P = *0.0498 for 1 week, 1 month, and 3 months, respectively); however, the reductions for the two groups were not significantly different at the 6-month visit (*P* = 0.5277).

**Table 2:** Corneal Subbasal Nerve Density and Keratocyte Density.

	Time after surgery				
	preoperative	1 week	1 month	3 months	6 months
Corneal Subbasal Nerve Density (mean ± SD, µm/mm^2^)					
SMILE	10875.7±3556.9	3331.1±2183.5	3265.6±2758.6	3944.0±3475.7	4941.2±3366.8
Decrease§		7608.3±576.6*	7642.1±564.6 *	6736.0±644.0*	5588.8±618.9*
FS-LASIK	9696.3±3085.6	70.7±229.4	398.9±871.2	1179.1±1920.2	3644.1±2848.1
Decrease§		9431.0±531.8†	9316.7±527.8†	8375.8±552.6†	5874.6±567.3†
△Decrease§		1822.7±784.4‡	1674.7±772.3‡	1639.8±847.3‡	285.8±838.4
Pre-IF keratocyte density (mean ± SD, cells/mm^2^)					
SMILE	261.3±47.7	183.6±40.5	181.5±35.6	184.7±34.7	166.2±34.4
Decrease§		77.8±10.0*	81.2±9.9*	83.0±11.4*	96.1±10.5*
FS-LASIK	230±26.6	160.4±38.5	174.0±46.1	150.6±39.5	164.8±40.0
Decrease§		72.5±8.4†	57.6±8.3†	81.4±8.9†	71.9±9.0†
△Decrease§		−5.3±13.1	−23.6±12.9	−1.5±14.4	−24.2±13.8
Post-IF keratocyte density (mean ± SD, cells/mm^2^)					
SMILE	209.5±40.0	107.4±31.6	112.0±31.1	123.3±42.8	107.7±29.4
Decrease§		102.3±8.2*	96.7±8.1*	85.4±9.3*	98.8±8.6*
FS-LASIK	221.2±28.6	147.8±33.0	138.2±30.3	135.1±30.0	120.7±29.8
Decrease§		72.2±6.9†	84.5±6.8†	85.7±7.3†	104.4±7.4†
△Decrease§		−30.1±10.7‡	−12.2±10.6	0.38±11.8	5.6±11.3

Pre-IF keratocyte density: pre interface keratocyte density.

Post-IF keratocyte density: post interface keratocyte density.

△Decrease, difference in the decreases between the two groups.

* *P*<0.0001, significantly different from the preoperative values in the SMILE group.

† *P*<0.0001, significantly different from the preoperative values in the FS-LASIK group.

‡ *P*<0.05, significantly different decrease in the two groups.

§ estimated from the mixed model.

**Figure 1:**
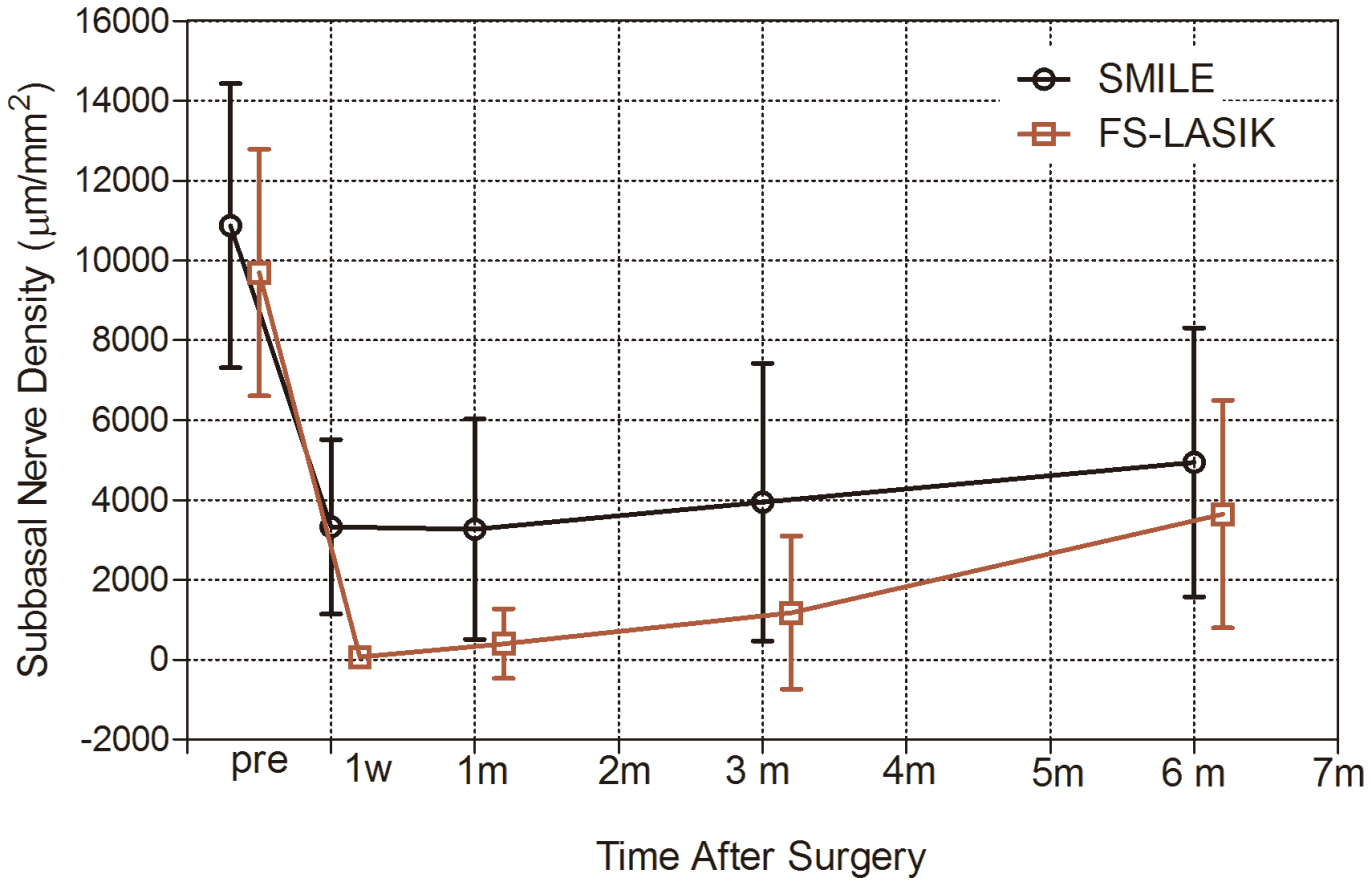
Recovery of subbasal nerve density after SMILE and FS-LASIK.

Graphing the results would be helpful. Figure 1 shows the changes in mean subbasal nerve density over time after SMILE and FS-LASIK. The postoperative corneal nerve fiber densities were decreased in both groups at all postoperative follow-up visits, with the greatest reduction evident at postoperative week 1. A gradual increase in corneal subbasal nerve density was observed throughout the follow-up.

### Recovery Patterns after SMILE and FS-LASIK

**Figure 2:**
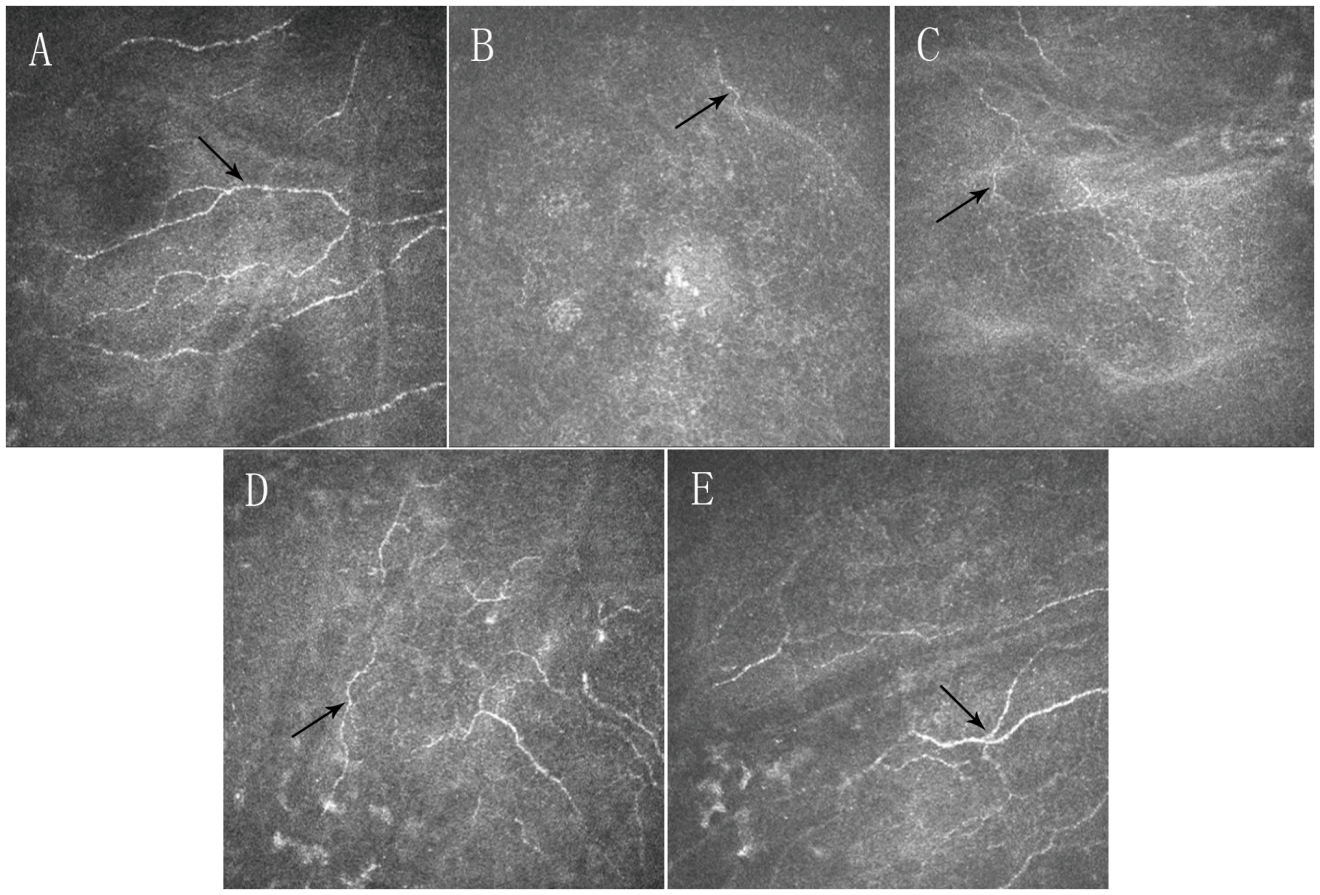
Confocal microscopy (HRT III) images showing time-dependent changes in the corneal subbasal nerve fibers in the central cornea in SMILE group. Preoperatively (A), intact corneal subbasal nerve fibers were noted (black arrow). At postoperative 1 week (B), 1 month (C), 3 months (D) and 6 months (E), subbasal nerve fibers were present in the central corneal area (black arrows). Postoperatively, the corneal subbasal nerve fibers appeared more tortuous compared to preoperative corneal subbasal nerve fibers (images acquired from the same subject). Field size: 400 µm×400 µm.

**Figure 3:**
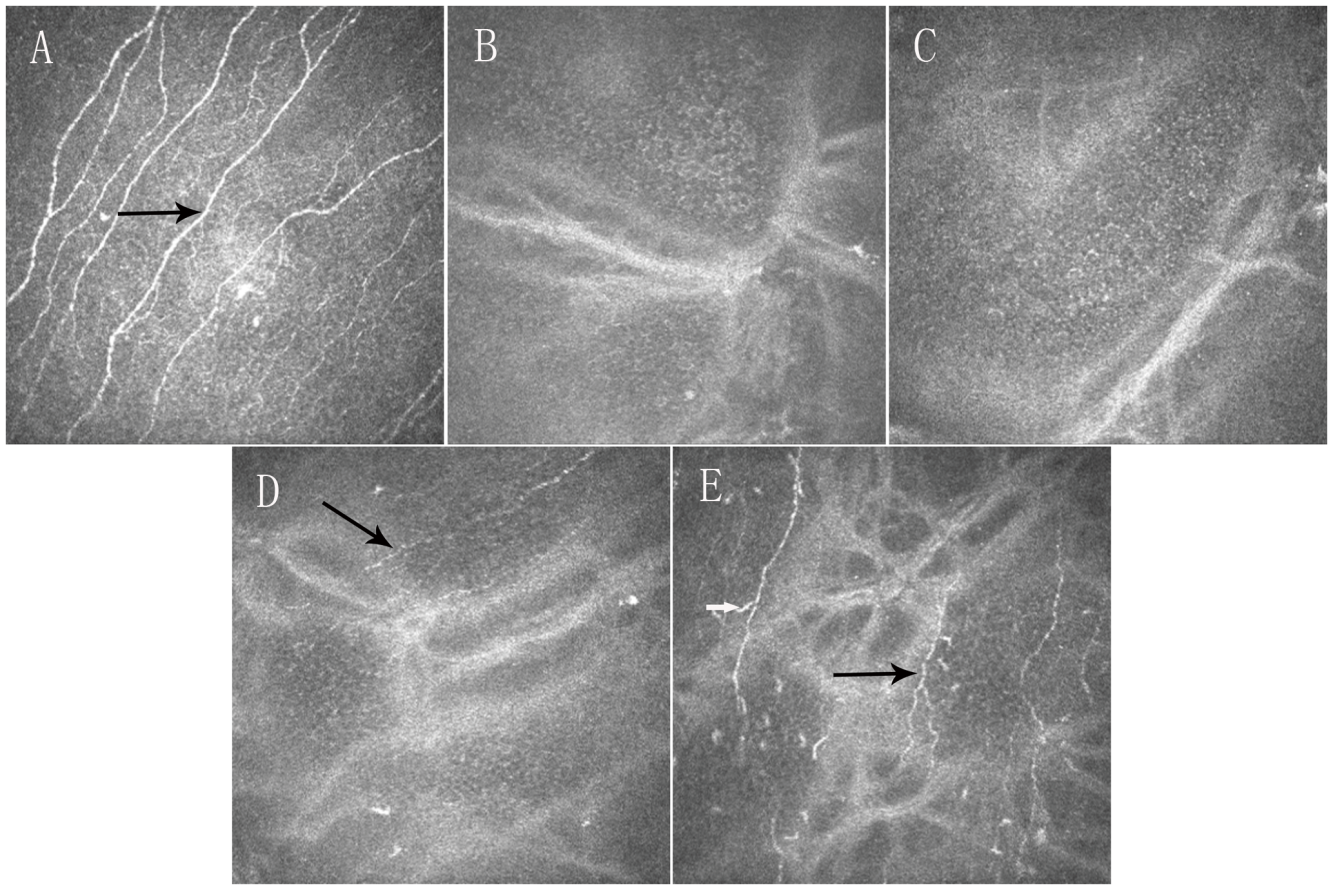
Confocal microscopy (HRT III) images showing time-dependent changes in the corneal subbasal nerve fibers in the central cornea in FS-LASIK group. Preoperatively (A), corneal nerves were evident with thick and regular appearance (black arrow). At postoperative 1-week (B) and 1-month visits (C), corneal subbasal nerve fibers were absent. At postoperative 3-month (D) and 6-month visits (E), subbasal nerve fibers were evident (black arrow). Note subbasal nerve fibers should be distinguished from dentric cells (white arrow) (images acquired from the same subject). Field size: 400 µm×400 µm.

The corneal subbasal nerve fibers in the corneal central region were detectable in 100% (29/29), 93.7% (30/32), 90.5% (19/21), and 100% (25/25) of SMILE-treated eyes at postoperative week 1, month 1, month 3, and month 6, respectively; (Figure 2) as compared to 15.2% (5/33), 29.4% (10/34), 56.6% (17/30), and 100% (28/28) respectively in FS-LASIK-treated eyes. (Figure 3) The regenerated nerve fibers appeared thin and tortuous compared with preoperative nerve fibers as seen in Figure 2 and Figure 3.

**Figure 4:**
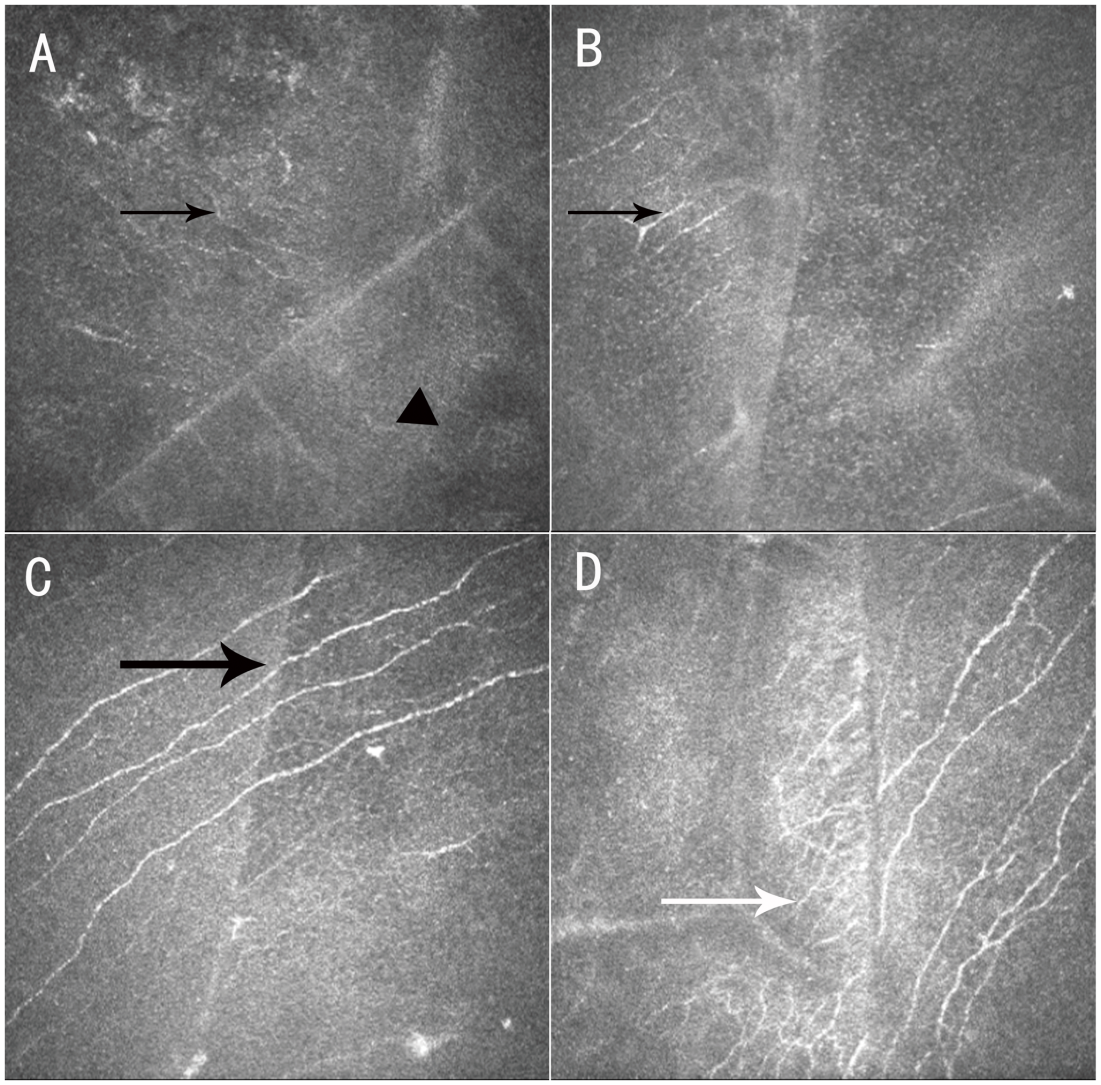
Confocal microscopy (HRT III) images showing nerve regeneration at the flap margins after FS-LASIK. At postoperative 1-week visit (A), subbasal nerve fibers were only noted outside the flap (black arrow), but not inside the flap (▴). At the 1-month visit (B), discrete subbasal nerve fibers were visible within the flap area (black arrow). At the 3-month visit (C), obvious subbasal nerve fibers with thick and regular appearance were seen (black arrow). At the 6-month visit (D), a lot of small-caliber nerve fibers with obvious tortuosity and loops were noted inside the flap (white arrow) (images acquired from the same subject). Field size: 400 µm×400 µm.

Nerve fiber regeneration at the flap margin usually originates from the peripheral cornea outside of the flap and moves to the denervated flap in FS-LASIK-treated eyes (Figure 4). Interestingly, one eye exhibited corneal subbasal nerve fibers that appeared thick and regular across the flap, and appeared to be reconnected perfectly at the two transected edges (Figure 4C).

### Keratocyte Density

Results of mixed-model tests showed that the decreases were statistically significant in both groups (Table 2). Additionally, the decrease in post-IF keratocyte density in the SMILE group was significantly higher than that in the FS-LASIK group at the 1-week visit (*P = *0.0055). However, the reductions for the two groups were not significantly different at any other postoperative follow-up visits (Table 2).

**Figure 5:**
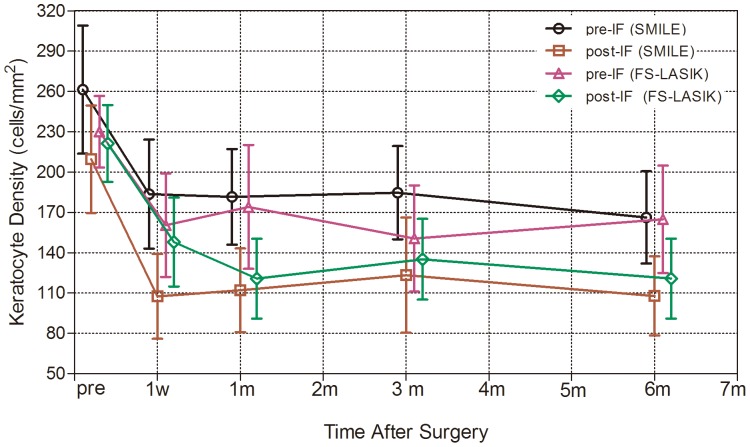
Time-dependent changes in the keratocyte density over time.

In order to visualize the trends of changes in keratocyte density, we graphed the mean keratocyte densities in Figure 5. The pre-IF keratocyte density and post-IF keratocyte density in both groups immediately decreased after surgery and showed no signs of recovery through the follow-up.

### Periphery of the Cap and Flap

**Figure 6:**
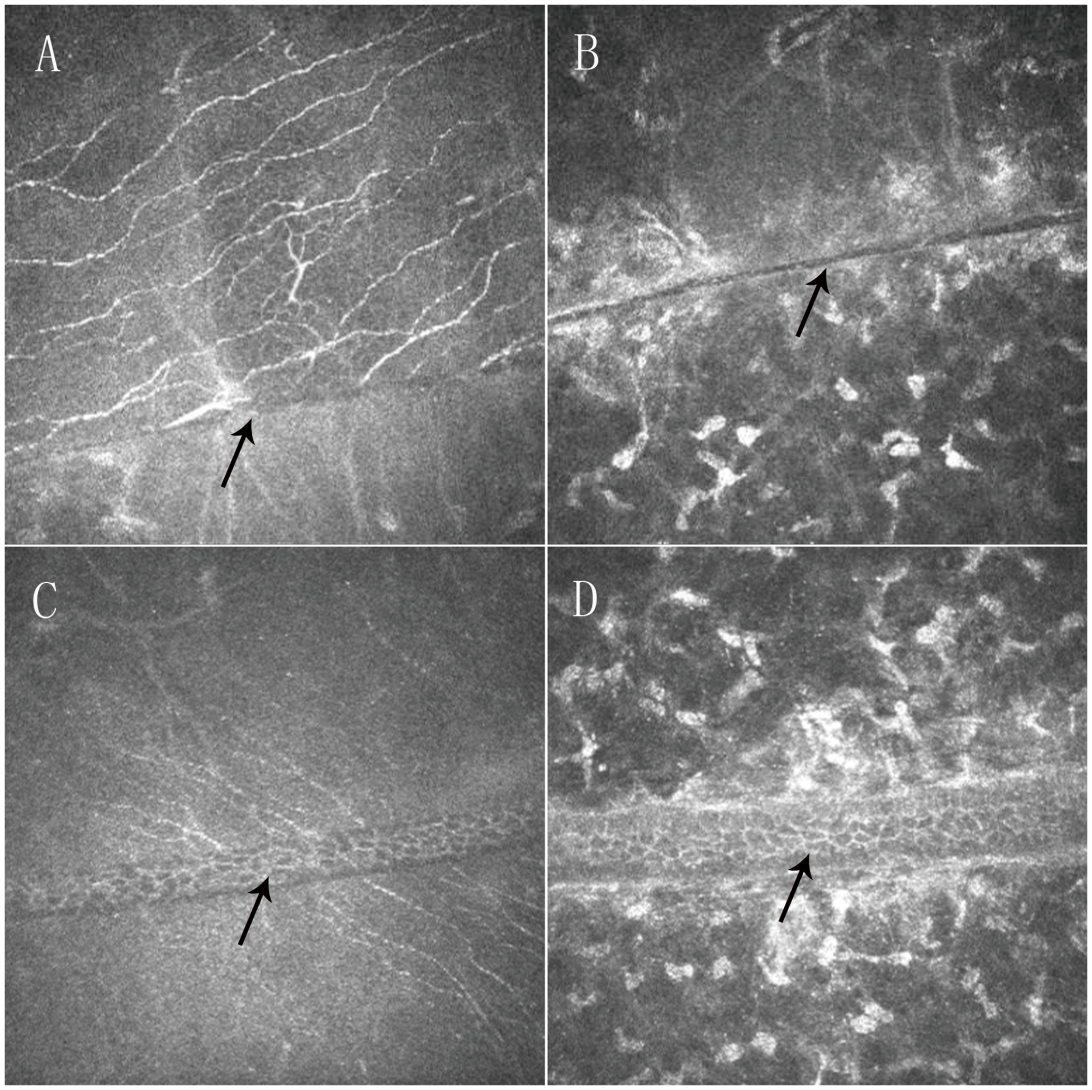
Confocal microscopy (HRT III) images showing clear and well-defined cap margins (A, B) and flap margins (C, D) 6 months postoperatively. Epithelial cells (arrows) were observed at the flap margins (C, D). The space between the margins was wider in FS-LASIK-treated eyes than in SMILE-treated eyes. Field size: 400 µm×400 µm.

Theoretically, a femtosecond laser is capable of creating precise cap and flap margins. This property was confirmed by confocal microscopic examination. The cap and flap margins were clear and well-defined as seen in Figure 6. At the 6-month visit, 52% (13/25) of SMILE-treated eyes and 85.7% (18/21) of FS-LASIK-treated eyes (*P* = 0.0005, Chi-square test) showed epithelial cells filling in the periphery empty space. Nine of the 18 eyes in the FS-LASIK group exhibited more than one peripheral area with epithelial cells filling in periphery empty space. Furthermore, the peripheral spaces tended to be wider in the FS-LASIK-treated eyes than in the SMILE-treated eyes (Figure 6).

### Correlations Analysis

The corneal sensitivity of the two groups is shown in Table 3. There was no statistically significant difference in baseline between the two groups (*P*>0.05). The corneal sensation in both groups was significantly decreased after surgery. The mean reduction in corneal sensation was less severe in the SMILE group than that in the FS-LASIK group.

**Table 3:** Corneal Sensitivity at Each Visit (mean ± SD).

	Time after surgery				
	preoperative	1 week	1 month	3 months	6 months
Corneal sensitivity					
SMILE	58.2±4.5	23.2±14.6	28.4±13.9	34.2±15.7	43.7±11.7
Decrease§		35.5±2.3*	30.4±2.3*	23.5±2.4*	12.9±2.4*
FS-LASIK	58.0±3.8	15.4±7.9	15.8±9.5	25.6±15.1	36.4±16.1
Decrease§		42.9±2.0†	42.5±2.1†	32.0±2.0†	21.4±2.1†
△Decrease§		7.4±3.1‡	12.1±3.1‡	8.5±3.1‡	8.5±3.2‡

△Decrease, difference in the decreases between the two groups.

* *P*<0.001, significantly different from the preoperative values in the SMILE group.

† *P*<0.01, significantly different from the preoperative values in the FS-LASIK group.

‡ *P*<0.05, more significant decrease in the FS-LASIK group than in the SMILE group.

§ estimated from the mixed model.

Spearman’s rank correlation test showed a moderate correlation between the subbasal nerve density and corneal sensitivity in the SMILE group (r = 0.416, *P*<0.0001) and a mild correlation in the FS-LASIK group (r = 0.2567, *P* = 0.0038). Neither pre-IF keratocyte density (r = −0.050, *P* = 0.626 in the SMILE group; r = 0.119, *P* = 0.189 in the FS-LASIK group) nor post-IF keratocyte density (r = 0.091, *P* = 0.370 in the SMILE group; r = −0.227, *P* = 0.051 in the FS-LASIK group) was correlated with subbasal nerve density.

## Discussion

Many studies have investigated corneal reinnervation after corneal refractive surgery for myopia correction. Patel *et al.* [13] demonstrated that the postoperative subbasal nerve densities observed 12 months after FS-LASIK and conventional LASIK were significantly lower than the preoperative densities. Zhang *et al.* [14] reported that corneal subbasal nerve fibers were detectable about 3 mm from the center of the flap in 62.5% of FS-LASIK-treated eyes at the 3-month visit and 100% of FS-LASIK-treated eyes at the 6-month visit. Consistent with previous studies, our results indicated that corneal subbasal nerve fibers were detectable in 56.6% of FS-LASIK-treated eyes at postoperative month 3 and in 100% of FS-LASIK-treated eyes at postoperative month 6. Vestergaard *et al*. [11] reported that the mean reduction in subbasal nerve density was 9.21±7.80 mm/mm^2^ at postoperative month 6 and suggested that the SMILE technique appeared better at sparing the central corneal nerves as compared to FLEX. In accordance with Vestergaard *et al*.’s [11] result, in the present study a statistically significant reduction was found in the mean subbasal nerve density (5588.8±618.9 µm/mm^2^) at postoperative month 6 in SMILE-treated eyes. Additionally, the greatest reduction in corneal subbasal nerve density was observed at postoperative week 1, and a slow increase was observed throughout the follow-up.

There are multiple factors to explain why the SMILE procedure preserved subbasal nerves at higher density in the 3 months after surgery. The fact that fewer corneal nerve fibers were transected in the SMILE procedure than in the FS-LASIK procedure may play an important role. Most of the corneal nerves are derived from the ophthalmic branch of the trigeminal nerve. [15] The majority of human stromal corneal nerves are located in the anterior 1/3 of the stroma and pierce the Bowman layer to form the subbasal nerve plexus in a radial fashion, extending from the periphery to the corneal center. [13] During the SMILE procedure, the incision was made in the superior quadrant with a circumferential length of 4.0∼4.5 mm; thus, a large portion of the anterior stromal nerves in the cap was not severed. In contrast, all of the superficial corneal nerve fibers, except the fibers at the hinge position, were transected in the FS-LASIK procedure. This was consistent with our findings that the central subbasal nerve fibers were detectable in nearly 100% of SMILE-treated eyes at all postoperative visits but were not detectable in FS-LASIK-treated eyes. But at the 6-month visit, no statistical difference was found in subbasal nerve density between the two groups. It is possible that other factors may have an effect on corneal reinnervation, such as the trophic substance, nerve growth factor (NGF). For example, Lee *et al.* [16] found differences in NGF levels in tears between LASIK and PRK-treated eyes in the early postoperative period, and the postoperative NGF concentration seemed to correlate with decreased corneal sensitivity. Thus, tear NGF may play a role in regulating corneal reinnervation after refractive surgeries. But further studies are needed to elucidate where NGF contributes to the different corneal reinnervation processes after SMILE vs FS-LASIK.

The morphological recovery patterns of corneal innervation did not differ postoperatively between SMILE and FS-LASIK. Neurites originating from the ends of the severed stromal nerves must pass through the denervated corneal cap or flap from the periphery to the center before re-establishing a new subbasal nerve plexus, [12] and the regenerated nerves are usually tortuous and thin. In one FS-LASIK-treated eye, the corneal subbasal nerve fibers across the flap appeared thick and regular, as if the fibers had never been severed (Figure 4). This finding might be explained by the precise realignment of proximal and distal Schwann cell channels. This indicates that good realignment of the cap or flap periphery could facilitate the re-connection or regrowth of severed nerve ends, and nerve cut-edges re-connection may be another repair pattern.

It has been considered that the loss of keratocyte density was due to keratocyte necrosis and apoptosis following the surgeries. The pre-IF keratocyte density and post-IF keratocyte density in both groups decreased immediately after surgery and did not show a sign of recovery. Consistently, Erie *et al* [17], [18] reported a persistent decrease in keratocyte density in the retroablation zone after LASIK. To date, the role of stromal keratocytes in the maintenance of cornea health and the clinical significance of the loss of keratocytes are still largely unknown. Our data showed no correlation between stromal keratocyte density and subbasal nerve density. Yet, Lee *et al*. [16] found that there was a strong correlation between subbasal nerve density and keratocyte density in their prospective LASIK and PRK series. The reasons for this discrepancy may be related to the different corneal layers focused in the two studies (Lee’s study: from the 131 µm to the Descemet membrane), and the different methods used for cell density calculations.

In the present study, subbasal nerve density was found to be positively correlated with corneal sensitivity, a finding that was partly consistent with the findings of Lee et *al.* [16], who reported a strong correlation between subbasal nerve density and corneal sensitivity after LASIK (r = 0.930, *P*<0.001) and laser epithelial keratomileusis (LASEK) (r = 0.678, *P*<0.001). In contrast, Patel *et al.* [13] did not detect a correlation between subbasal nerve density and corneal sensitivity after LASIK, nor did Darwish *et al.* [19]. This discrepancy may be due to the use of different confocal devices, which may be less sensitive to detect the regeneration of small-caliber nerves [13].

SMILE-treated eyes are less likely to have peripheral spaces that require epithelial cells to fill in, and the spaces that are present tend to be smaller than those observed in FS-LASIK-treated eyes. The clinical consequences of the presence or absence of epithelial cells at the cap or flap margins are unknown. Whether this property will lead to an increased risk of epithelial cell invasion or weak flap adhesion is unclear. But we speculate that smaller peripheral spaces may facilitate the re-connection of severed nerve ends, as shown in Figure 4 C.

In summary, this study for the first time confirmed that the decrease in subbasal nerve density was less severe in the SMILE group than the FS-LASIK group and subbasal nerve density was positively correlated with corneal sensitivity. In addition, this study is the first to report that SMILE-treated eyes had a lower risk of epithelial cells filling in empty marginal spaces than FS-LASIK-treated eyes, which may facilitate corneal reinnervation as well as the nerve cut-edges re-connection. This study provides important data for the understanding of the corneal reinnervation process after refractive surgeries and helps to guide further clinical refinement of corneal refractive surgeries.
